# Multi-year tracking reveals extensive pelagic phase of juvenile loggerhead sea turtles in the North Pacific

**DOI:** 10.1186/s40462-016-0087-4

**Published:** 2016-10-03

**Authors:** D. K. Briscoe, D. M. Parker, S. Bograd, E. Hazen, K. Scales, G. H. Balazs, M. Kurita, T. Saito, H. Okamoto, M. Rice, J. J. Polovina, L. B. Crowder

**Affiliations:** 1 Hopkins Marine Station, Stanford University, Pacific Grove, CA USA; 2 Joint Institute for Marine and Atmospheric Research, National Oceanic and Atmospheric Administration, Newport, OR USA; 3 Environmental Research Division, National Marine Fisheries Service, National Oceanic and Atmospheric Administration, Southwest Fisheries Science Center, Monterey, CA USA; 4 National Marine Fisheries Service, National Oceanic and Atmospheric Administration, Pacific Islands Fisheries Science Center, Honolulu, HI USA; 5 Port of Nagoya Public Aquarium, Minato-ku, Nagoya, 455-0033 Japan; 6 Usa Marine Biological Institute, Kochi University, Usa Tosa, Kochi, 781-1164 Japan; 7 Hawaii Preparatory Academy, 65-1692 Kohala Mt. Rd. Kamuela, Hawaii, 96743 USA; 8 Center for Ocean Solutions, Stanford University, Monterey, CA USA

**Keywords:** Loggerhead sea turtle, Migration, Foraging, Movement, Distribution, Pelagic

## Abstract

**Background:**

The juvenile stage of loggerhead sea turtles (*Caretta caretta*) can last for decades. In the North Pacific Ocean, much is known about their seasonal movements in relation to pelagic habitat, yet understanding their multi-year, basin-scale movements has proven more difficult. Here, we categorize the large-scale movements of 231 turtles satellite tracked from 1997 to 2013 and explore the influence of biological and environmental drivers on basin-scale movement.

**Results:**

Results show high residency of juvenile loggerheads within the Central North Pacific and a moderate influence of the Earth’s magnetic field, but no real-time environmental driver to explain migratory behavior.

**Conclusions:**

We suggest the Central North Pacific acts as important developmental foraging grounds for young juvenile loggerhead sea turtles, rather than just a migratory corridor. We propose several hypotheses that may influence the connectivity between western and eastern juvenile loggerhead foraging grounds in the North Pacific Ocean.

**Electronic supplementary material:**

The online version of this article (doi:10.1186/s40462-016-0087-4) contains supplementary material, which is available to authorized users.

## Background

Highly mobile marine species utilize dynamic oceanographic habitats as they move between breeding and non-breeding habitats [1, 2]. While advancements in tracking have greatly enhanced our ability to understand how migratory animals move through their environment (seabirds, marine mammals, and sea turtles), it still remains a challenge to understand the degree of connectivity between the reproductive and foraging grounds [3]. Environmental flows such as wind and currents are known to influence early animal movement into the open ocean, and can potentially impact the ontogeny of foraging and migratory patterns as animals develop [4–6]. However, limitations still exist in our ability to track individuals throughout life history stages [7]. For many species, the pelagic stage is inferred rather than empirically observed [8]. This is especially true for sea turtles, as the oceanic period of early life history has been termed the ‘lost years’ [9].

**Fig. 1:**
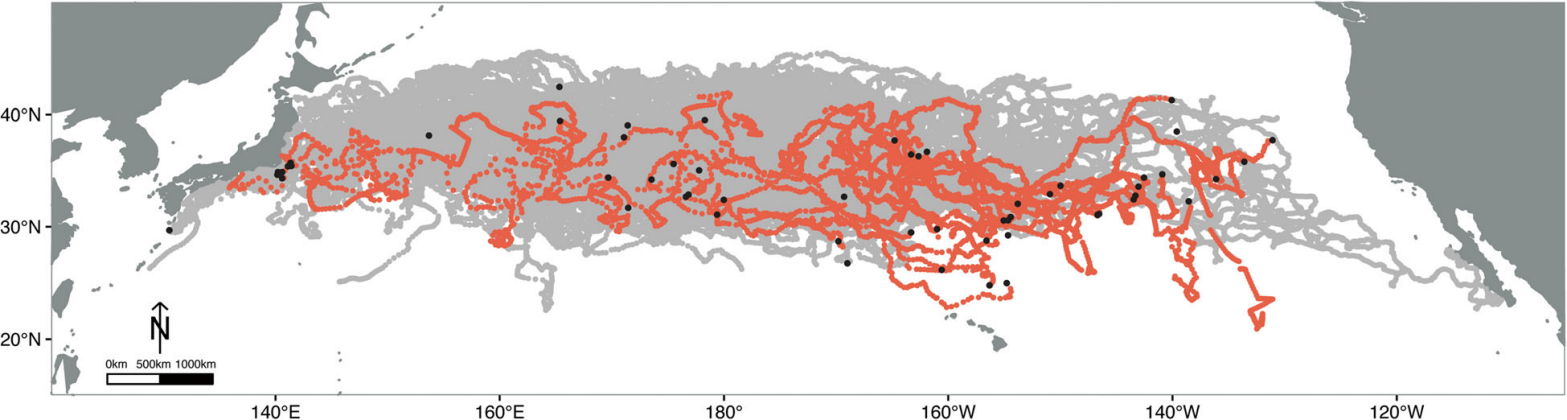
Map of the North Pacific Ocean and 231 satellite tagged juvenile loggerhead sea turtle locations from 1997 to 2014. Gray tracks represent 197 captive-reared juveniles; red tracks represent wild-caught juveniles. Black dots indicate the deploy locations for individual tracks

The long-term tracking data of juvenile 231 loggerhead sea turtles (*Caretta caretta*) provides an unprecedented opportunity to examine the large-scale movements and distribution of individuals during a poorly understood life history stage (Fig. 1). In the North Pacific, hatchlings leave their natal beaches of Japan and undergo a multi-year migration within the North Pacific Gyre [10, 11]. Juveniles are known to forage throughout the Central North Pacific (CNP) [12, 13], migrating to eastern developmental grounds, along the Baja California Peninsula, Mexico (BCP) [14, 15]. Upon reaching maturity, turtles migrate back to their natal beaches of Japan and remain in the western Pacific as adults [11, 16, 17]. Studies of this population have provided exceptional insight into seasonal foraging movements, diet, and active dispersal of juveniles throughout the North Pacific [12, 13, 18–25].

Because the juvenile life history stage can last up to three decades [26–28], the timing, duration, and long-term patterns of movement across the ocean basin still remain relatively unknown [29]. As a result, there remain important gaps in our understanding of the basin-scale movements and residence time within foraging habitats, in particular the connectivity between juvenile foraging grounds, which impedes conservation efforts along migratory routes [30]. Here, we sought to: (1) explore movements and connectivity between nesting and foraging grounds of juvenile North Pacific loggerhead sea turtles, and (2) explore the influence of biological and environmental drivers on basin-scale movements. Results show a lengthy residence time of juveniles within the Central North Pacific. These results challenge the long-standing belief that juvenile loggerheads use the Central North Pacific as a migratory corridor to eastern basin developmental grounds. The extended time within the open ocean reveals many individuals move back and forth within the pelagic environment for several years.

## Methods

### Animal tracking

Our study involves the synthesis of 186 previously published tracks [12, 13, 18–21, 24, 25, 31] and includes 45 new tracks from 2011 to 2013 (Table 1). This is the first publication of all tracks from 1997 to 2013. Briefly, we review the outfitting procedure. One hundred and ninety-seven sea turtles were hatched and raised in the Port of Nagoya Public Aquarium in Minoto-ku, Japan. Thirty-four were wild-caught juveniles. Individuals were released in the ocean in several locations off of Japan or in the Central North Pacific (CNP) (Fig. 1a). Argos-linked satellite transmitters were attached to the carapace of all juveniles, following the procedures recommended in [32]. Argos-derived surface locations were collected by the NOAA PIFSC, Marine Turtle Research Program, Honolulu, Hawaii.

**Table 1:** Summary of 231 satellite tracked juvenile loggerhead sea turtles in the North Pacific Ocean

Deploy Date	Deploy E Lon	Deploy N Lat	# Deployed	Captive/Wild Caught	SCL (cm)	Age at Deploy (months)	Days Transmitted	Distance Traveled (km)
24-Apr-03	140.2	34.6	7	Captive	38.9–59.4	20–44	67–565	1939–12,357
28-Nov-03	140.2	34.9	17	Captive	26.2–56	15–40	48–1270	2517–22,415
19-Nov-04	140.6	34.9	26	Captive	27.7–35.3	15	85–464	3263–11,832
19-Apr-05	141.1	35.4	12	Captive	25.6–64.8	24–48	27–617	873–16,338
4-May-05	176.6	32.7	40	Captive	29.6–38.4	20	229–1434	3852–25,900
27-Oct-06	176.8	32.9	34	Captive	24.4–29.5	NA	47–493	1258–8075
31-Mar-09	141.4	35.4	16	Captive	30.9–37.2	19	97–696	3044–15,196
9-Apr-10	130.5	29.7	17	Captive	32.8–40.7	20	268–558	5937–12,226
12-Jul-11	141.3	35.7	15	Captive	34.5–71.1	24–60	11–480	275–11,112
12-Jul-11	153.7–180.0	31.7–42.4	13	Captive	34.9–39.1	24	571–865	11,425–17,863
23-Jan-97	169.8	28.7	1	Wild	44.5	NA	55	1013
2-Feb-97	163.3	29.5	1	Wild	52	NA	115	2592
15-Feb-97	161.0	29.8	1	Wild	41	NA	90	1311
17-Mar-97	154.4	30.9	1	Wild	62	NA	136	3480
30-Mar-97	160.6	26.2	1	Wild	73	NA	42	989
10-Apr-97	169.0	26.7	1	Wild	73.6	NA	13	372
20-Apr-97	154.7	29.2	1	Wild	53.7	NA	12	295
22-Apr-97	156.6	28.8	1	Wild	81	NA	178	5199
11-Sep-97	131.1	37.7	1	Wild	45	NA	67	1703
6-Jan-98	143.0	33.6	1	Wild	45.5	NA	206	3136
7-Jan-98	142.5	34.4	1	Wild	48	NA	191	3518
7-Feb-98	154.7	30.6	1	Wild	58	NA	103	1876
7-Feb-98	155.1	30.5	1	Wild	61	NA	71	1442
26-Aug-98	162.6	36.3	1	Wild	58	NA	167	2301
26-Aug-98	163.3	36.4	1	Wild	57.7	NA	106	2001
18-Oct-98	164.8	37.7	1	Wild	52.5	NA	41	935
20-Oct-98	139.6	38.5	1	Wild	59.1	NA	161	2448
2-Nov-98	161.9	36.7	1	Wild	62.5	NA	51	737
10-Dec-98	136.1	34.2	1	Wild	56.5	NA	6	13
23-Dec-98	210.0	33.6	1	Wild	57.5	NA	211	4426
31-Jan-99	156.3	24.8	1	Wild	83	NA	51	1024
3-Feb-99	153.8	32.0	1	Wild	52.5	NA	131	1727
14-Dec-99	209.1	32.9	1	Wild	51.5	NA	271	5180
17-Jan-00	143.3	32.8	1	Wild	62	NA	72	1766
3-Feb-00	169.3	32.7	1	Wild	67	NA	157	4221
12-Feb-00	138.6	32.3	1	Wild	55	NA	49	1177
5-Mar-00	146.6	31.1	1	Wild	60	NA	597	13,864
7-Mar-00	146.7	31.1	1	Wild	56	NA	246	3757
30-May-00	154.8	25.0	1	Wild	83	NA	138	3543
19-Aug-00	226.4	35.8	1	Wild	61	NA	177	3237
14-Oct-02	140.1	41.3	1	Wild	55.5	NA	358	9591
25-Dec-02	219.1	34.7	1	Wild	45.5	NA	245	4464
7-Jan-03	216.5	32.4	1	Wild	43.5	NA	226	3800
1-Aug-03	140.6	34.3	1	Wild	68.1	NA	336	14,579

Sea turtles were deployed within two regions: the Western North Pacific (Japan) and the Central North Pacific (CNP)

All raw surface locations were filtered and regularized using a Bayesian State Space Switching Model (SSSM). Developed by [33], the SSSMs account for observation error, in order to regularize animal location estimates in time, as well as interpolate over small gaps resulting from missing observations of animals locations [1]. Final position estimates along each track were generated at 24-h intervals.

### Characterization of basin-scale movements

**Fig. 2:**
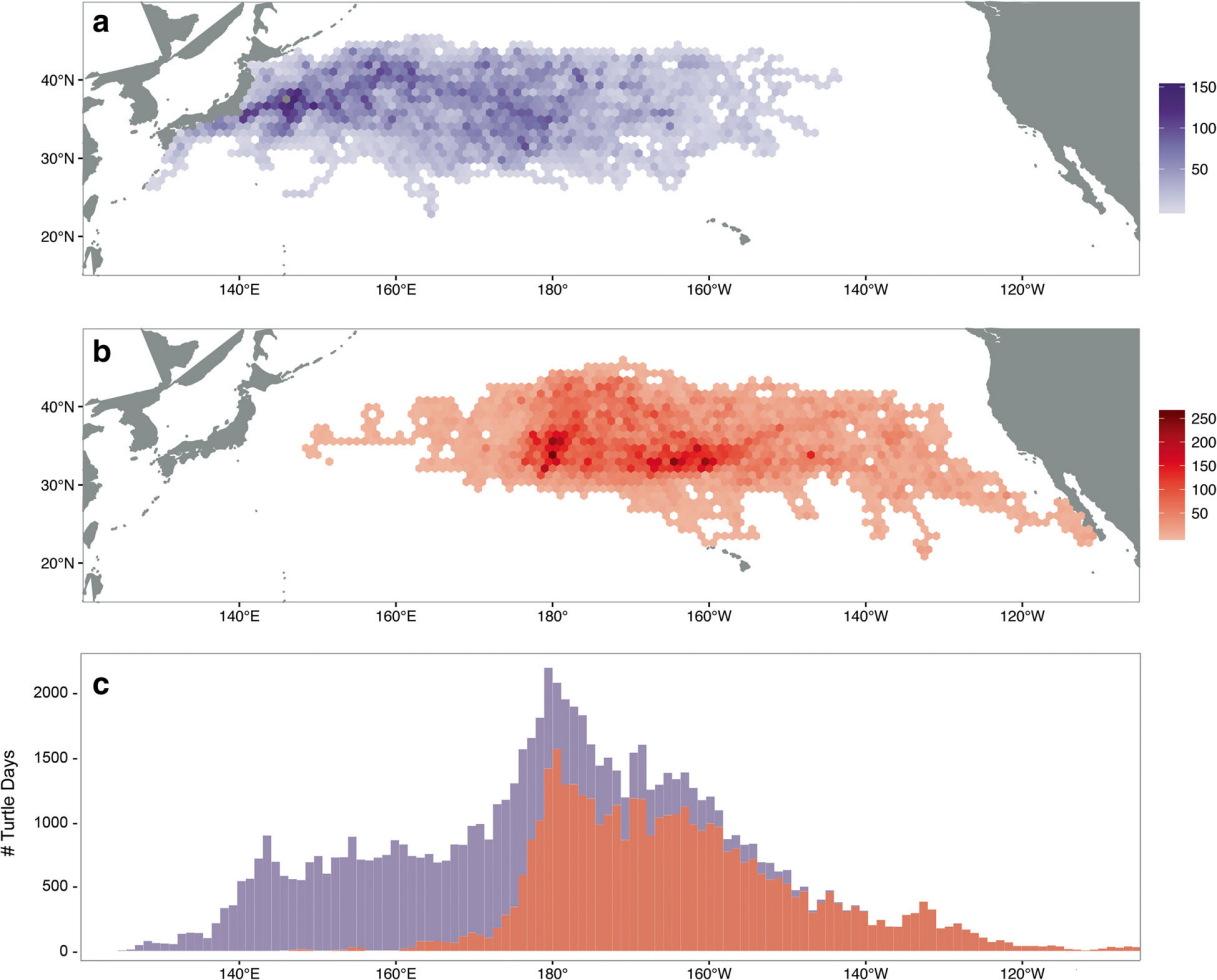
Spatial use of juvenile loggerhead sea turtles, color-coded by deploy location: **a** Japan (*purple*) and (**b**) the Central North Pacific (*red*). The number of turtle days are represented by each 1° hexagonal bin. **c** Frequency use by degree longitude, color-coded by deploy region: Japan (*purple*) and the Central North Pacific (*red*)

**Fig. 3:**
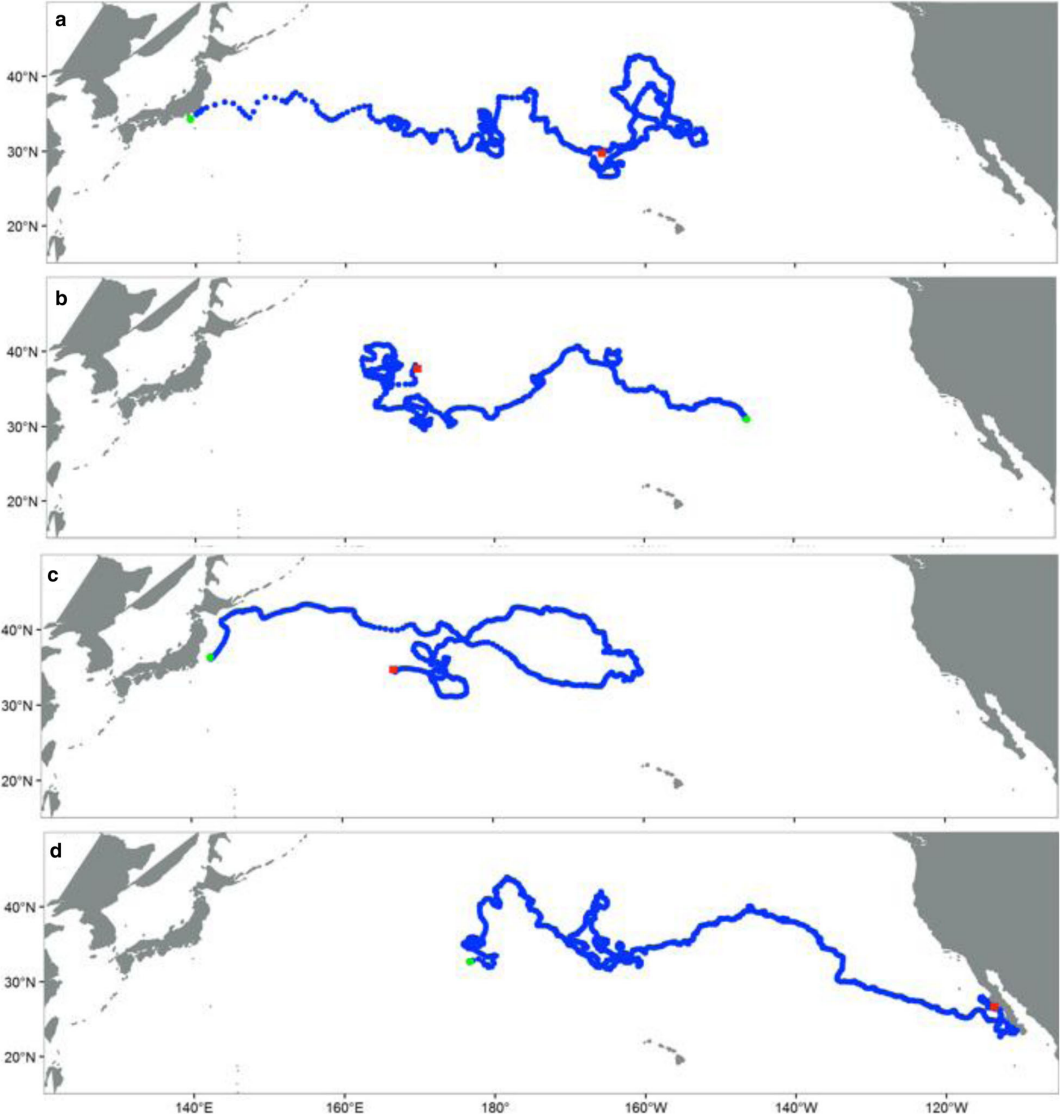
Example tracks showing the dominant migratory routes from these data: **a** moving eastward within the CNP, **b** moving westward within the CNP, **c** moving east then west, staying within the CNP, and **d** moving eastward to Baja California, Mexico. Track deploy locations are in green and end locations are in red

**Fig. 4:**
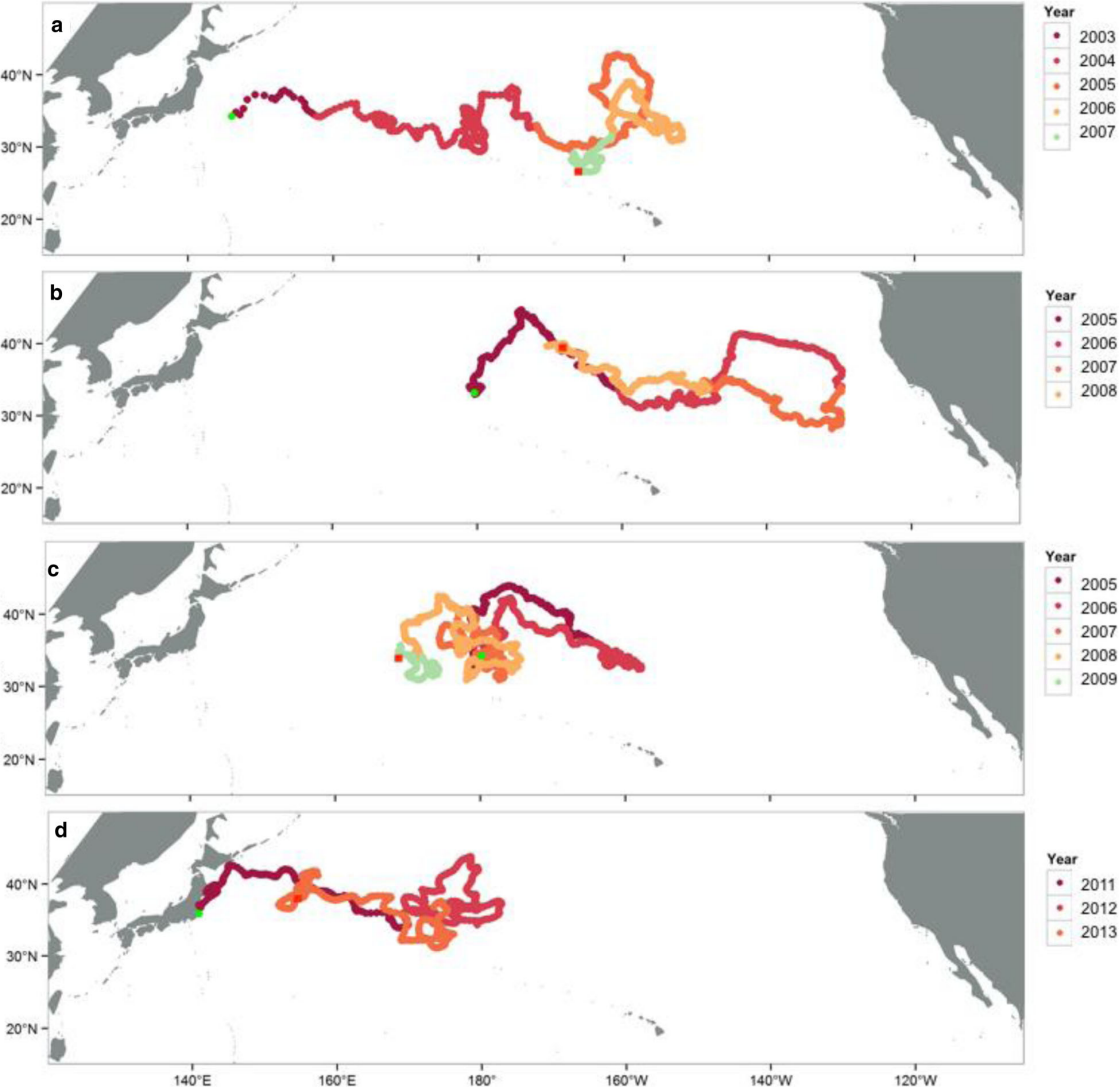
Example tracks showing the high residence time within the Central North Pacific (CNP). Tracks are color-coded by year: **a** Track ‘23045’, number of days transmitted: 1270; **b** Track ‘50136’, number of days transmitted: 1247; **c** Track ‘57148’, number of days transmitted: 1434; and **d** Track ‘23002’, number of days transmitted: 865. Track deploy locations are in green and end locations are in red

In order to characterize long-term, large-scale movements of juvenile loggerheads in the North Pacific, we applied several filters to account for tagging bias and deploy location, and the presence of short or incomplete tracks [34, 35]. Tracks were first categorized by deploy region (Japan or CNP). Individuals that transmitted for less than 60 days were removed from the data set. From these data, spatial density maps were used to calculate areas of high residency, based on deploy location. Using hexagonal polygon binning, we calculated the number of days spent in a 1° longitudinal area, similar to [36] (Fig. 2). The movements of each individual track were classified into one of three dominant migratory routes, based on direction of travel. Throughout all tracks, the seasonal north-south migration with the Transition Zone Chlorophyll Front (TZCF) is evident, as described by [31]. Because we have long-term, multi-year data for these animals, we can go beyond seasonal movements to look at the larger, basin scale movements of their migrations. For this reason, movements were categorized by their east-west dynamics: (1) moving eastward within the CNP foraging grounds (e.g. Fig. 3a), (2) moving westward within the CNP (e.g. Fig. 3b), (3) moving eastward but underwent a considerable change in direction (e.g. moving east then west, or ‘turning around’, thereby staying within the CNP) (e.g. Fig. 3c), and (4) moving eastward to Baja California, Mexico foraging grounds (e.g. Fig. 3d). Since almost all tracks displayed portions of eastward migration (Fig. 2), tracks that moved east then west were identified by a subsequent longitudinal displacement of at least 3° in the westward direction (Fig. 2c).

### Exploration of environmental and biological drivers of basin-scale movements

#### Remotely-sensed oceanographic data

Remotely sensed environmental data were obtained for each loggerhead track location using Xtractomatic (http://coastwatch.pfel.noaa.gov/xtracto/). The data sets included time-series of SST, surface chlorophyll-a concentrations, geostrophic current components (u and v), and SST variability (i.e. SST root mean square, SST RMS) (Additional file 1: Table S1). For each oceanographic parameter, a mean value was calculated based on the mean latitude and longitude error (0.1° longitude × 0.1° latitude × (1 to 8 day intervals) and centered at the position of each daily SSSM-interpolated turtle position (*sensu* [37]). Transformations of the parameters were explored to ensure data were normally distributed. A logarithmic transformation was required for chlorophyll-a. A square root transformation was applied to SST RMS.

#### IGRF magnetic field data

Geomagnetic data were available from NOAA’s National Geophysical Data Center (NGDC; http://www.ngdc.noaa.gov/geomag). Magnetic field observations were calculated using the International Geomagnetic Reference Field (IGRF) coefficients. As the Earth’s magnetic field changes over time, these coefficients were updated by the International Association of Geomagnetism and Aeronomy to accurately reflect the magnetic field at present (NGDC 2015, link above). The most recent, 11th generation coefficients for total magnetic intensity, inclination, and declination were used in this study, as each have been shown to be detectable by sea turtles [25, 38–42]. A 1° by 1° monthly grid of each field component was calculated for the North Pacific Ocean basin using the GeoMag 7.0 software available from the NGDC.

Generalized Additive Models (GAMs) were used to explore the environmental and biological factors associated with sea turtles that moved eastward across the North Pacific but then reversed direction, staying within the CNP (Fig. 3c, Additional file 1: Table S2).

## Results

### Track movements

Two hundred and thirty-one juvenile loggerhead sea turtles (23.3–83 SCL cm) were tracked from January 1997 – November 2013. One-hundred and ten aquarium-reared and 24 wild-caught individuals were released off of Japan. Seventy-four aquarium-reared and 23 individuals were released within the CNP. Deployments ranged from 41 to 1434 days (mean 351 days ± 256 days SD). Of these, 95 individuals transmitted for greater than 1 year; 14 for more than 2 years and 8 for more than 3 years.

Individuals deployed off Japan utilized the Kuroshio Extension Current (KEC) to disperse into pelagic areas (Fig. 2a). Individuals deployed within the CNP showed high use between 180° and 160°W (Fig. 2b). Mapping of loggerhead movements showed that irrespective of deploy location, turtles displayed an extended residence time (greater than 100 turtle days per grid cell), between 165°E–158°W longitudes (Fig. 2c). This long-term residency was demonstrated by several individuals that traversed back and forth within the CNP for several years (Fig. 4).

One hundred and forty-seven individuals (63.6 % of the total) displayed an eastward only migratory pattern before end of transmission (376.2 days ± 252.3 days), moving an average distance of 2028 km (±2454.3 km). Of these, ten were wild-caught turtles.

Nineteen sea turtles (8.2 % of the total), all wild-caught, moved westward from their CNP deploy locations, towards Japan (mean 199.6 days ± 192.9 days, 3105 km ± 2096.3 km). Sixty-five individuals (28.1 % of the total) moved eastward and then reversed direction along their migration routes, staying within the CNP (582 days ± 304.7 days, mean 11,128 km ± 4939.3 km). Five were wild-caught individuals. Thirty-four of these 65 turtles were deployed off of Japan and reached an average maximum eastward longitude of 180° before reversing direction (Additional file 1: Figure S1). The remaining 31 turtles were deployed throughout the Central North Pacific and reached an average maximum longitude of 160°W (Additional file 1: Figure S1). Only one out of all 231 turtles migrated to Baja California (Fig. 3d).

### Environmental data

**Fig. 5:**
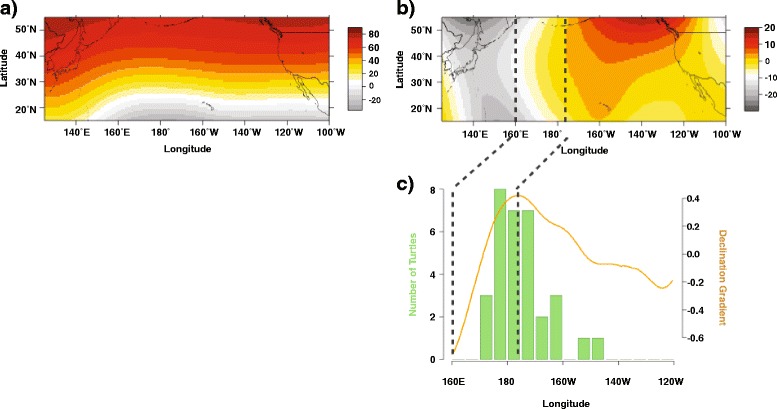
IGRF-10 Earth magnetic field values for (**a**) inclination and (**b**) declination, across the North Pacific Ocean basin; (**c**) Number of turtles that reversed direction (staying within the CNP) by longitude (*green*). Overlain in orange is the average change in declination by longitude (gradient). The sharpest change in declination occurs between 160°E - 180° longitude (*dashed lines*). This region is known for its biological productivity [13]

Median sea surface temperatures experienced by turtles was 17.6 °C (±2.2 °C, range of 10.0–28.74 °C) and 0.2 °C (±0.2 °C, range of 0–1.9 °C) for SST RMS. Median concentration of chlorophyll-a was 0.3 mg m^-3^ (±1.8 mg m^-3^, range of 0.0–72.8 log mg m^-3^). Median magnetic field values were 51.3° (±5.1°, range of 26.3–62.1°) for inclination, -0.1° (±5.0°, range of -16.1–6.3°) for declination, and 43,820 nT (±1749.2 nT, range of 35,640–49,410 nT) for total intensity. Sea turtles deployed off of Japan experienced the greatest change in the Earth’s magnetic field declination, corresponding to 160° E - 180° (Fig. 5b-c). Individuals that were deployed within the CNP were deployed eastward of this gradient, and thus did not experience the same regional change in the Earth’s Magnetic declination as the individuals deployed off of Japan.

### GAM results

No real-time environmental variables were significant in understanding a change in direction for the 65 individuals that moved from east to west (Additional file 1: Table S3). The two variables that were primarily attributed to a change in direction for the western Pacific deployed turtles were magnetic field declination (Fig. 5 and the number of days traveled (Additional file 1: Figure S4a-b)). For the central North Pacific, the two variables most correlated to a change in direction were magnetic field inclination and the month. These models revealed that western Pacific deployed turtles are more likely to continue traveling eastward under lower magnetic field declination values and for the first 250–300 days of travel, whereas the CNP turtles are more likely to continue traveling eastward under higher values of magnetic field inclination and towards the latter half of the year (Fig. 5 and Additional file 1: Figure S4c-d).

## Discussion

This study combined information from two decades of satellite tracking of juvenile loggerhead sea turtles in the North Pacific to describe the movements of individuals during a poorly understood life history stage. Results show a long-term residence of sea turtles within the Central North Pacific and an exceptional level of variability in their individual migration strategies across the ocean basin. Contrary to expectations, we found no real-time environmental influences to explain migratory behavior. However, a moderate influence of the Earth’s magnetic field was detected, suggesting that movements may be driven by the navigational markers that help guide them towards thermally optimal and biologically favorable habitats within the open ocean, similar to Atlantic loggerhead sea turtles [40].

Traditional loggerhead sea turtle life history assumed that young, at-sea turtles were passive migrants, transported cross-basin along migratory corridors to eastern boundary foraging grounds [9]. Upon reaching these developmental grounds, juveniles were thought to undergo an ontogenetic shift from oceanic to neritic habitat [10]. Currents may carry individuals to suitable places that they revisit as juveniles, thereby shaping the ontogenetic development of migration routes [43]. Indeed, recent studies in both the Atlantic and Pacific Ocean basins have suggested that active dispersal and orientation play larger than expected roles in the at-sea movements of young loggerheads [22, 44]. In this study, both wild-caught and captive-reared individuals moved in eastward and westward directions across the ocean basin, suggestive that it may be part of the natural behavior of individuals from this population to move back and forth within the CNP. However, of the 231 juvenile loggerheads tracked up to 4.9 years, only one underwent a successful migration to Baja California (Fig. 1a). This is perplexing, as the coastal waters of the BCP in the eastern North Pacific are believed to be an important developmental foraging ground for the entire population [15]. Conditions off of BCP in the east are more energetically efficient including faster growth rates and eventually higher fecundity, albeit with the potential trade-off of higher predation risk [28]. Recent estimates suggest that up to 43,000 juveniles utilize this foraging hotspot each year [15]. Based on these results, we propose several hypotheses that may explain why juveniles may stay within the CNP for extended periods of time, instead of migrating directly to the Baja California Peninsula.

### Hypothesis 1: CNP juveniles are not mature enough to recruit to BCP

To date, most studies of foraging loggerheads off the Baja California coast have focused on large juveniles (55–85 SCL cm) [28, 45]. For this reason, it could be suggested that turtles from this data set were too young to undergo an ontogenetic shift to neritic waters. However, recent skeletochronology has aged BCP turtles as young at 3 years old, which overlaps with ages of turtles tracked in this study [46]. This matches the age and size range of the one turtle from this data set that did migrate to the BCP. It should be noted that this turtle was originally deployed in the Central North Pacific, essentially giving it a head start towards its eastern foraging grounds. However, [28] found there to be no significant difference in the SCL sizes of CNP and BCP juveniles.

### Hypothesis 2: Some utilize pelagic waters throughout their entire juvenile life history stage

Because loggerheads have been shown to take advantage of both oceanic and neritic habitats as both juveniles and subadults [47, 48], it is entirely possible that not all loggerhead turtles in the North Pacific undergo trans-Pacific dispersals, but instead use pelagic habitat for their entire juvenile phase, as suggested by [13]. A recent study of a subset of individuals from this population showed active orientation of juveniles within the CNP [22]. For this reason, it may be that the CNP is not just a migratory corridor that juveniles pass through on the way to foraging grounds off Baja California, but representative of juvenile foraging grounds altogether. The extended residence time within the CNP and lack of a migration to the BCP could be indicative of an alternative life history strategy for juveniles of this population [28].

### Hypothesis 3: Turtles returning to previously experienced preferable habitat

For many migratory species (birds, tuna, sharks, and other species of sea turtles), complete migrations are not simple or direct [34, 49–51]. In fact, indirect routes are ‘followed not to only avoid unfavorable areas but as a pragmatic solution to completing a long journey successfully’ [50]. This may be especially true for early stage sea turtles, as they are free from the constraints of breeding, and are able to seek out the most productive areas to optimize growth while avoiding thermal stressors [28, 52]. Long distance migration across the CNP is likely energetically costly. Upon leaving the seasonally productive waters of the Kuroshio Extension Bifurcation Region (KEBR) and the Transition Zone Chlorophyll Front (TZCF) (*see* [13, 31], foraging opportunities in the eastern CNP may be increasingly difficult.

That turtles frequently appear to turn around as they move further east across the North Pacific and after a mean of 334.5 (±227.8 SD) days at sea, may be due to reaching a maximum energetic threshold (see [50]). Such a detour, or re-entry, of juveniles into the biologically productive waters of the KEBR and TZCF would allow turtles to minimize their energetic costs of travel, that prevents fasting and allows turtles to refuel for a potentially long-distance migration to the eastern Pacific developmental grounds. Similar behavior has been shown for green turtles (*Chelonia mydas*) in the South Atlantic, as they undergo transoceanic migrations between breeding sites (Ascension Island) and coastal foraging sites along Brazil [50].

Just how loggerheads may be able to successfully navigate a return to foraging hotspots has been better understood in other populations. In the North Atlantic Ocean, extensive research has shown that early stage loggerheads display a versatile navigational system. Namely, their open ocean migration is guided in part by passive drift associated with North Atlantic Subtropical Gyre (NASG) and Gulf Stream circulation, and active orientation due to swimming [9, 47, 53, 54]. There is a strong selective pressure for a juvenile turtle to remain within geographic areas that provide suitable conditions [55]. Poor navigation outside the gyre could lead to lethal temperatures [41]. This is done by active orientation in relation to a regional magnetic map [39–42, 54, 56]. These studies have effectively shown that the transoceanic migration of loggerheads in the North Atlantic, and the geographic regions they utilize along their migratory path, including ontogenetic shifts in habitat—are in large part bounded by the navigational markers associated with changes in the magnetic field [40–42]. In the North Pacific, magnetic influences have not been studied as in depth, however [25] noted that changes in the magnetic field could influence habitat choice on a basin-scale level.

Results from this study show that, similar to North Atlantic studies, North Pacific juveniles may use the Earth’s magnetic field [41] to reorient themselves back to the favorable habitat of the KEBR, while staying within thermally suitable latitudes. The isoclines of magnetic field inclination are similar to the latitudinal changes in SST (Fig. 5a and Additional file 1: Figure S3a). As individuals move with the north-south trend of the TZCF [20, 31], isoclines of inclination may help prevent animals from being swept by currents into inhospitable temperatures, also similar to the North Atlantic [41]. Turtles deployed off Japan travel through the Kuroshio Extension Current and Bifurcation Region (KEC and KEBR, respectively), and thus, a sharp gradient in declination as they make their way through the CNP, from 160°E - 180° longitude (Fig. 5b-c). This is the same region known for its biological productivity [13] (Additional file 1: Figure S3b). Several studies have examined the potential for magnetic maps to be ‘imprinted’ upon turtles throughout their transoceanic migrations [43, 57, 58]. Therefore, one explanation for a reversal in migration is the use of indirect movements based on the number of days traveled and the increase in energetic costs. As turtles move eastward throughout the CNP, foraging opportunities may be less prevalent, triggering them to turn around and return to more favorable habitat using a combination of regional navigational markers.

### Hypothesis 4: Migration routes may be tied to genetics

Until the 1990s, the origins of Baja California loggerheads were entirely unknown. Work by [10] connected the haplotypes of Baja juveniles to observed haplotypes of loggerheads found off Japan. It is now known that nesting for the entire North Pacific loggerhead population is restricted to the Japanese Archipelago [10, 17, 59].

More recent work has begun to highlight significant differentiation among Japanese rookeries [11, 60] and their influences on the spatial distribution of feeding aggregations, suggesting that genetic composition of loggerheads may be tied to post-nesting migration patterns [61]. We hypothesize that these genetic differences may also express themselves during developmental migrations, such that there may be a genetic component from some nesting beaches that contribute to Baja California migrations. Despite the large sample size, it is possible that satellite tagged animals from this study representative of nesting beaches that do not display this genetic contribution. However, until there is better resolution of the genetics of juvenile North Pacific loggerheads, this hypothesis will remain difficult to elucidate.

### Hypothesis 5: Environmental conditions influence recruitment to BCP

Like all ectotherms, sea turtles are inherently tied to the temperature of their surrounding environment. Studies have shown that higher water temperatures are energetically more favorable for sea turtles in terms of growth, digestion, and maintenance of core body temperature, up to a 30 °C thermal maxima [14, 62, 63]. Moreover, several management strategies currently use SST and ENSO events as a metric of bycatch avoidance for the North Pacific loggerhead population (*see* [24, 64]). During El Nino years, i.e. when SSTs are anomalously warmer, the California Drift Gillnet Fishery is closed due to increased interaction with loggerhead sea turtles [64]; an event that is not experienced during other oceanographic regimes. It is possible that interannual variability and/or anomalous SSTs play a larger role in east-west movements, promoting or prohibiting movement eastwards, towards Baja California, Mexico. However, more data is needed to fully explore this hypothesis.

### Caveats

It should be noted that there are some caveats to these data, which may hinder our ability to fully understand the relationship between loggerhead movement and their environment. Daily SST measurements may capture mesoscale features, but may not be representative of relationships to anomalous events or larger-scale oceanographic and atmospheric variability (e.g. ENSO). The absence of a prey field and intermittent satellite data due to cloud cover (i.e. chlorophyll concentration) may prevent the model from being more robust. Ultimately, models that incorporate an index of forage and that address turtle energetics may be needed to further advance our understanding of movement. Further, the majority of the tracks represent captive-reared turtles (*n* = 204 out of 231 turtles). While some caution must be taken in comparing captive with wild caught turtle behavior, no study to date has observed significant differences in migration and swimming behavior between the two [13, 21]. However, further studies are needed to compare the habitat and behaviors of captive-reared and wild-caught sea turtles under similar environmental conditions [21].

## Conclusions

North Pacific loggerhead sea turtles travel long distances and across entire ocean basins to reach developmental foraging grounds, yet connectivity between east-west movements has remained difficult. While individuals from this endangered population display a range of movement strategies, results from multi-year tracking reveals extensive use of the Central North Pacific. Here, we have shown that east-west movements may be due to environmental cues in the Earth’s magnetic field, which may aid in navigation back to preferable habitat. We suggest the Central North Pacific acts as important developmental foraging grounds for young juvenile loggerhead sea turtles, rather than just a migratory corridor, as results of this study show that many potentially important areas utilized by oceanic loggerheads may fall within unprotected areas of the high seas, offering critical geographic information that may be used for spatially-explicit conservation approaches within the pelagic environment. Further understanding the movement ecology of juvenile North Pacific loggerheads is therefore crucial for more efficient conservation strategies of this population.

## Additional file

Additional file 1: Table S1. Satellite product and spatio-temporal resolution of environmental variables sampled underneath of loggerhead tracks. **Table S2.** Summary of the 65 satellite tracked juvenile loggerhead sea turtles in the North Pacific Ocean that displayed turn around behavior. Turtles were deployed within two regions: the Western North Pacific (Japan) and the Central North Pacific (CNP). **Table S3.** Model selection results from generalized additive modeling of the environmental conditions at the location of reversal in migration (‘turnaround’) versus the conditions experienced by a turtle as it continued to move eastward across the North Pacific Ocean. Presented are the p-values, r-squared, estimated degrees of freedom, and AIC for each of the eleven environmental parameters for (a) Japan deployed turtles and (b) turtles deployed within the Central North Pacific. **Figure S1.** Density histogram of maximum eastward longitude for (a) all turtles, *n* = 231 (gray), long-term tracks deployed off Japan, *n* = 34 (blue line), and long-term deployments in the Central North Pacific, *n* = 31 (green line). **Figure S2.** Examples of east-west movement in individual long-term tracks. Example of 2 turtles deployed in the western (a) and (b) central North Pacific that reached a maximum eastward trajectory and changed dominant direction. Panels c and d show each track moving in an east-west-east direction, with the start location designated by a blue triangle and final location designated with a red square. Both tracks initially moved eastward (gray line) and then reversed direction, moving westward (blue segment of track). Both changed dominant direction for second time, once again moving eastward (green segment of track). Track ‘68330’ transmitted for 614 days (15,289 km). Track ‘22534’ transmitted for 1047 days (18,238 km). **Figure S3.** (a) Average sea-surface temperature (SST °C) and (b) Chlorophyll-a concentrations (mg m^-3^) for the North Pacific Ocean basin, from 1997 to 2014). **Figure S4.** GAM response curves of juvenile loggerhead east-west movements. Positive values show the likelihood of an animal reversing direction under a range of environmental values. Results indicate that turtles deployed off Japan are more likely to reverse direction with an increase in declination (a) and the longer it travels since deployment (b). For the turtles deployed in the Central North Pacific, turtles are more likely to reverse direction under lower values of magnetic field inclination (c) and during the first half of the year (d). Gray shading represents the 95 % confidence intervals for the fitted relationships. (DOCX 1198 kb)

## References

[1] Block BA, Jonsen ID, Jorgensen SJ, Winship AJ, Shaffer SA, Bograd SJ, Hazen EL, Foley DG, Breed GA, Harrison AL (2011). Tracking apex marine predator movements in a dynamic ocean. Nature.

[2] Hays GC, Ferreira LC, Sequeira AM, Meekan MG, Duarte CM, Bailey H, Bailleul F, Bowen WD, Caley MJ, Costa DP (2016). Key questions in marine megafauna movement ecology. Trends Ecol Evol.

[3] Godley BJ, Barbosa C, Bruford M, Broderick AC, Catry P, Coyne MS, Formia A, Hays GC, Witt MJ (2010). Unravelling migratory connectivity in marine turtles using multiple methods. J Appl Ecol.

[4] Gaspar P, Georges J-Y, Fossette S, Lenoble A, Ferraroli S, Le Maho Y (2006). Marine animal behaviour: neglecting ocean currents can lead us up the wrong track. Proc Roy Soc B.

[5] Chapman JW, Klaassen RH, Drake VA, Fossette S, Hays GC, Metcalfe JD, Reynolds AM, Reynolds DR, Alerstam T (2011). Animal orientation strategies for movement in flows. Curr Biol.

[6] Scott R, Hays GC (2014). Ontogeny of long distance migration. Ecology.

[7] Hazen EL, Jorgensen S, Rykaczewski RR, Bograd SJ, Foley DG, Jonsen ID, Shaffer SA, Dunne JP, Costa DP, Crowder LB, Block BA (2012). Predicted habitat shifts of Pacific top predators in a changing climate. Nat Climate Change.

[8] Varo-Cruz N, Bermejo JA, Calabuig P, Cejudo D, Godley BJ, López-Jurado LF, Pikesley SK, Witt MJ, Hawkes LA, Roura-Pascual N. New findings about the spatial and temporal use of the Eastern Atlantic Ocean by large juvenile loggerhead turtles. Divers Distributions. 2016;22:481-92.

[9] Carr A (1987). New perspectives on the pelagic stage of sea turtle development. Conserv Biol.

[10] Bowen B, Abreu-Grobois F, Balazs G, Kamezaki N, Limpus C, Ferl R (1995). Trans-Pacific migrations of the loggerhead turtle (Caretta caretta) demonstrated with mitochondrial DNA markers. Proc Natl Acad Sci U S A.

[11] Hatase H, Kinoshita M, Bando T, Kamezaki N, Sato K, Matsuzawa Y, Goto K, Omuta K, Nakashima Y, Takeshita H (2002). Population structure of loggerhead turtles, Caretta caretta, nesting in Japan: bottlenecks on the Pacific population. Mar Biol.

[12] Parker DM, Cooke WJ, Balazs GH (2005). Diet of oceanic loggerhead sea turtles (Caretta caretta) in the central North Pacific. Fish Bull.

[13] Polovina J, Uchida I, Balazs G, Howell EA, Parker D, Dutton P (2006). The Kuroshio Extension Bifurcation Region: A pelagic hotspot for juvenile loggerhead sea turtles. Deep Sea Res II.

[14] Peckham SH, Maldonado-Diaz D, Koch V, Mancini A, Gaos A, Tinker MT, Nichols WJ, Wallace J (2008). High mortality of loggerhead turtles due to bycatch, human consumption and strandings at Baja California Sur, Mexico, 2003 to 2007. Endang Species Res.

[15] Seminoff JA, Eguchi T, Carretta J, Allen CD, Prosperi D, Rangel R, Gilpatrick JW, Forney K, Peckham SH (2014). Loggerhead sea turtle abundance at a foraging hotspot in the eastern Pacific Ocean: implications for at-sea conservation. Endang Species Res.

[16] Nichols W, Resendiz A, Seminoff J, Resendiz B (2001). Transpacific migration of a loggerhead turtle monitored by satellite telemetry. Bull Mar Sci.

[17] Kamezaki N, Matsuzawa Y, Abe O, Asakawa H, Fujii T, Goto K, Hagino S, Hayami M, Ishii M, Iwamoto T: Loggerhead turtles nesting in Japan. Loggerhead Sea Turtles. 2003:210-217.

[18] Polovina JJ, Balazs GH, Howell EA, Parker DM, Seki MP, Dutton PH (2004). Forage and migration habitat of loggerhead (Caretta caretta) and olive ridley (Lepidochelys olivacea) sea turtles in the central North Pacific Ocean. Fish Oceanog.

[19] Polovina JJ, Howell E, Parker DM, Balazs GH (2003). Dive-depth distribution of loggerhead(Carretta carretta) and olive ridley(Lepidochelys olivacea) sea turtles in the Central North Pacific: Might deep longline sets catch fewer turtles?. Fish Bull.

[20] Polovina JJ, Kobayashi DR, Parker DM, Seki MP, Balazs GH (2000). Turtles on the edge: movement of loggerhead turtles (Caretta caretta) along oceanic fronts, spanning longline fishing grounds in the central North Pacific, 1997–1998. Fish Oceanog.

[21] Abecassis M, Senina I, Lehodey P, Gaspar P, Parker D, Balazs G, Polovina J (2013). A model of loggerhead sea turtle (Caretta caretta) habitat and movement in the oceanic North Pacific. PLoS One.

[22] Briscoe D, Parker D, Balazs G, Kurita M, Saito T, Okamoto H, Rice M, Polovina J, Crowder L. Active dispersal in loggerhead sea turtles (Caretta caretta) during the ‘lost years’. In Proc R Soc B. The Royal Society. 2016;283:20160690. 10.1098/rspb.2016.0690 PMC4920322 27252021

[23] Howell EA, Dutton PH, Polovina JJ, Bailey H, Parker DM, Balazs GH (2010). Oceanographic influences on the dive behavior of juvenile loggerhead turtles (Caretta caretta) in the North Pacific Ocean. Mar Biol.

[24] Howell EA, Kobayashi DR, Parker DM, Balazs GH, Polovina a (2008). TurtleWatch: a tool to aid in the bycatch reduction of loggerhead turtles Caretta caretta in the Hawaii-based pelagic longline fishery. Endangered Species Res.

[25] Kobayashi DR, Polovina JJ, Parker DM, Kamezaki N, Cheng IJ, Uchida I, Dutton PH, Balazs GH (2008). Pelagic habitat characterization of loggerhead sea turtles, Caretta caretta, in the North Pacific Ocean (1997–2006): Insights from satellite tag tracking and remotely sensed data. J Exp Mar Biol Ecol.

[26] Snover ML. Growth and ontogeny of sea turtles using skeletochronology: methods, validation and application to conservation. PhD Thesis, Duke University; 2002.

[27] Heppell S, Crowder L, Crouse D, Epperly S, Frazer NB (2003). Population models for Atlantic loggerheads: past, present, and future.

[28] Peckham SH, Maldonado-Diaz D, Tremblay Y, Ochoa R, Polovina J, Balazs G, Dutton PH, Nichols WJ (2011). Demographic implications of alternative foraging strategies in juvenile loggerhead turtles Caretta caretta of the North Pacific Ocean. Mar Ecol Prog Ser.

[29] Okuyama J, Kitagawa T, Zenimoto K, Kimura S, Arai N, Sasai Y, Sasaki H (2011). Trans-Pacific dispersal of loggerhead turtle hatchlings inferred from numerical simulation modeling. Mar Biol.

[30] Ikeda T (2008). Seasonal Distribution and Behavior of Loggerhead Sea Turtles in the North Pacific: statistical analysis in relation to environmental oceanographic parameters.

[31] Polovina JJ, Howell E, Kobayashi DR, Seki MP (2001). The transition zone chlorophyll front, a dynamic global feature defining migration and forage habitat for marine resources. Prog Oceanogr.

[32] Balazs GH, Miya RK, Beavers S (1996). Procedures to attach a satellite transmitter to the carapace of an adult green turtle, Chelonia mydas. NOAA Tech Memo NMFS SEFSC.

[33] Jonsen ID, Myers RA, James MC (2006). Robust hierarchical state-space models reveal diel variation in travel rates of migrating leatherback turtles. J Anim Ecol.

[34] Perle CR. Movements and Migrations of Manta Rays, Pacific Bluefin Tuna, and White Sharks: Observations and Insights at the Intersection of Life History and Ecosystem Structure. Stanford University, 2011.

[35] Winship AJ, Jorgensen SJ, Shaffer SA, Jonsen ID, Robinson PW, Costa DP, Block BA (2012). State‐space framework for estimating measurement error from double‐tagging telemetry experiments. Methods Ecol Evol.

[36] Witt MJ, Augowet Bonguno E, Broderick AC, Coyne MS, Formia A, Gibudi A, Mounguengui Mounguengui GA, Moussounda C, NSafou M, Nougessono S (2011). Tracking leatherback turtles from the world’s largest rookery: assessing threats across the South Atlantic. Proc Biol Sci.

[37] Shillinger GL, Swithenbank AM, Bailey H, Bograd SJ, Castelton MR, Wallace BP, Spotila JR, Paladino FV, Piedra R, Block BA (2011). Vertical and horizontal habitat preferences of post-nesting leatherback turtles in the South Pacific Ocean. Mar Ecol Prog Ser.

[38] Light P, Salmon M, Lohmann KJ (1993). Geomagnetic orientation of loggerhead sea turtles: evidence for an inclination compass. J Exp Biol.

[39] Fuxjager MJ, Eastwood BS, Lohmann KJ (2011). Orientation of hatchling loggerhead sea turtles to regional magnetic fields along a transoceanic migratory pathway. J Exp Biol.

[40] Lohmann KJ, Cain SD, Dodge SA, Lohmann CM (2001). Regional magnetic fields as navigational markers for sea turtles. Science.

[41] Lohmann KJ, Putman NF, Lohmann CM (2012). The magnetic map of hatchling loggerhead sea turtles. Curr Opin Neurobiol.

[42] Putman NF, Endres CS, Lohmann CM, Lohmann KJ (2011). Longitude perception and bicoordinate magnetic maps in sea turtles. Curr Biol.

[43] Hays GC, Fossette S, Katselidis KA, Mariani P, Schofield G (2010). Ontogenetic development of migration: Lagrangian drift trajectories suggest a new paradigm for sea turtles. J R Soc Interface.

[44] Putman NF, Mansfield KL (2015). Direct evidence of swimming demonstrates active dispersal in the sea turtle “lost years”. Curr Biol.

[45] Peckham SH, Maldonado Diaz D, Koch V, Mancini A, Gaos A, Tinker MT, Nichols WJ (2008). High mortality of loggerhead turtles due to bycatch, human consumption and strandings at Baja California Sur, Mexico, 2003 to 2007. Endangered Species Res.

[46] Tomaszewicz CN, Seminoff JA, Avens L, Goshe LR, Peckham SH, Rguez-Baron JM, Bickerman K, Kurle CM (2015). Age and residency duration of loggerhead turtles at a North Pacific bycatch hotspot using skeletochronology. Biol Conserv.

[47] Bolten AB (2003). Variation in sea turtle life history patterns: neritic vs. oceanic developmental stages. Biol Sea Turtles.

[48] McClellan CM, Read AJ (2007). Complexity and variation in loggerhead sea turtle life history. Biol Lett.

[49] Alerstam T (2001). Detours in bird migration. J Theor Biol.

[50] Hays G, Broderick A, Godley B, Lovell P, Martin C, McConnell B, Richardson S (2002). Biphasal long-distance migration in green turtles. Anim Behav.

[51] Espinoza M, Heupel MR, Tobin AJ, Simpfendorfer CA (2016). Evidence of partial migration in a large coastal predator: opportunistic foraging and reproduction as key drivers?. PLoS One.

[52] Stearns S (1996). The evolution of life histories.

[53] Scott R, Marsh R, Hays GC (2011). A little movement orientated to the geomagnetic field makes a big difference in strong flows. Mar Biol.

[54] Putman NF, Verley P, Shay TJ, Lohmann KJ (2012). Simulating transoceanic migrations of young loggerhead sea turtles: merging magnetic navigation behavior with an ocean circulation model. J Exp Biol.

[55] Shillinger GL, Di Lorenzo E, Luo H, Bograd SJ, Hazen EL, Bailey H, Spotila JR (2012). On the dispersal of leatherback turtle hatchlings from Mesoamerican nesting beaches. Proc Roy Soc B.

[56] Lohmann KJ, Lohmann CM (1996). Detection of magnetic field intensity by sea turtles. Nature.

[57] Lohmann KJ, Putman NF, Lohmann CM (2008). Geomagnetic imprinting: a unifying hypothesis of long-distance natal homing in salmon and sea turtles. Proc Natl Acad Sci.

[58] Gaspar P, Benson SR, Dutton PH, Réveillère A, Jacob G, Meetoo C, Dehecq A, Fossette S (2012). Oceanic dispersal of juvenile leatherback turtles: going beyond passive drift modeling. Mar Ecol Prog Ser.

[59] Bowen B, Karl S (2007). Population genetics and phylogeography of sea turtles. Mol Ecol.

[60] Watanabe KK, Hatase H, Kinoshita M, Omuta K, Bando T, Kamezaki N, Sato K, Matsuzawa Y, Goto K, Nakashima Y (2011). Population structure of the loggerhead turtle Caretta caretta, a large marine carnivore that exhibits alternative foraging behaviors. Mar Ecol Prog Ser.

[61] Nishizawa H, Narazaki T, Fukuoka T, Sato K, Hamabata T, Kinoshita M, Arai N (2014). Genetic composition of loggerhead turtle feeding aggregations: migration patterns in the North Pacific. Endangered Species Res.

[62] Bjorndal KA (1980). Nutrition and grazing behavior of the green turtle Chelonia mydas. Mar Biol.

[63] Coles W, Musick JA, Price A (2000). Satellite sea surface temperature analysis and correlation with sea turtle distribution off North Carolina. Copeia.

[64] Allen CD, Lemons GE, Eguchi T, LeRoux RA, Fahy CC, Dutton PH, Peckham SH, Seminoff JA (2013). Stable isotope analysis reveals migratory origin of loggerhead turtles in the Southern California Bight. Mar Ecol Prog Ser.

