# Supplementary material for: Multi-year tracking reveals extensive pelagic phase of juvenile loggerhead sea turtles in the North Pacific
Source: Mov Ecol. 2016 Oct 3;4:23. doi: 10.1186/s40462-016-0087-4 (PMC5048666; doi:10.1186/s40462-016-0087-4)
Supplement: Additional file 1: Table S1. — Satellite product and spatio-temporal resolution of environmental variables sampled underneath of loggerhead tracks. Table S2. Summary of the 65 satellite tracked juvenile loggerhead sea turtles in the North Pacific Ocean that displayed turn around behavior. Turtles were deployed within two regions: the Western North Pacific (Japan) and the Central North Pacific (CNP). Table S3. Model selection results from generalized additive modeling of the environmental conditions at the location of reversal in migration (‘turnaround’) versus the conditions experienced by a turtle as it continued to move eastward across the North Pacific Ocean. Presented are the p-values, r-squared, estimated degrees of freedom, and AIC for each of the eleven environmental parameters for (a) Japan deployed turtles and (b) turtles deployed within the Central North Pacific. Figure S1. Density histogram of maximum eastward longitude for (a) all turtles, n = 231 (gray), long-term tracks deployed off Japan, n = 34 (blue line), and long-term deployments in the Central North Pacific, n = 31 (green line). Figure S2. Examples of east-west movement in individual long-term tracks. Example of 2 turtles deployed in the western (a) and (b) central North Pacific that reached a maximum eastward trajectory and changed dominant direction. Panels c and d show each track moving in an east-west-east direction, with the start location designated by a blue triangle and final location designated with a red square. Both tracks initially moved eastward (gray line) and then reversed direction, moving westward (blue segment of track). Both changed dominant direction for second time, once again moving eastward (green segment of track). Track ‘68330’ transmitted for 614 days (15,289 km). Track ‘22534’ transmitted for 1047 days (18,238 km). Figure S3. (a) Average sea-surface temperature (SST °C) and (b) Chlorophyll-a concentrations (mg m-3) for the North Pacific Ocean basin, from 1997 to 2014). Figure S4. GAM response curves [file 40462_2016_87_MOESM1_ESM.docx]

**Briscoe et al. -- Supplemental Text**

*Generalized Additive Models (GAMs)*

Generalized Additive Models (GAMs) were used to investigate the environmental and biologic factors associated with a loggerhead moving eastward versus those experienced upon a significant change in direction (east-west) (Fig 3b and c). GAMs allow for multiple non-linear relationships between a response variable and its covariates in a semi-parametric manner [30-32]. Such models identify the distribution of the dependent parameter and apply an additive function to determine the variables’ response to predictors [33, 34].

A suite of explanatory variables was considered for the models, all of which have been previously explored as important features for oceanic loggerheads in the North Pacific [11, 13-16, 40]. These included environmental variables: sea-surface temperature (SST), chlorophyll-a, the three magnetic field components (inclination, declination, and total intensity), as well as biological: standard carapace length (SCL) at time of deployment, the number of days transmitted, distance traveled, and the month of turnaround.

*Data selection and model*

Because of the wide range in spatial deployments, separate models were run for each of the two deploy regions: Japan and Central North Pacific. A binary model with a negative binomial link function was used to compare the difference in environmental conditions experienced by a loggerhead moving eastward versus those conditions experienced as they reversed direction from east to west.

In order to avoid deploy location bias, no track locations were chosen along the first half of transmission, nor the 30 days prior to a major change in direction. For example, if a turtle transited eastward for 100 days upon deployment, and then changed dominant direction on day 100, 5 eastward location points were randomly chosen between day 50 and day 70. These locations were then compared with the environmental conditions along the 5 days before and after turning around (i.e. days 98-102) (SFig 2a-d). This 5-day window helped to (1) identify environmental conditions along a route change and (2) minimize potential outliers and/or gaps in environmental data due to cloud and satellite coverage.

Various combinations of the remaining data sets were included in the GAMs to determine the most robust model, using forward and backward stepwise model selection. Model robustness was compared using an information theoretic modeling approach. Explanatory variables included in the final model were chosen based on the model with the lowest Akaike’s Information Criterion (AIC) and highest receiver operating curve (ROC) area under the curve (AUC) statistics [41]. GAMs were run in R (version 3.1) using the mgcv package (version 1.7-6). The GAM model with the lowest AIC and highest AUC value was run 100 times with a 1:1 ratio of turnaround to randomly chosen eastward location for each tag, in order to examine its significance.

*GAM results*

The two variables that were primarily attributed to a change in direction for the western Pacific deployed turtles were magnetic field declination and the number of days traveled (SFig 4a-b). For the central North Pacific, the two variables most correlated to a change in direction were magnetic field inclination and the month. The models revealed that western Pacific deployed turtles are more likely to continue traveling eastward under lower magnetic field declination values and for the first 250-300 days of travel, whereas the CNP turtles are more likely to continue traveling eastward under higher values of magnetic field inclination and towards the latter half of the year (Fig 4a and SFig 4c-d).

**Supplemental Tables**

**STable 1.** Satellite product and spatio-temporal resolution of environmental variables sampled underneath of loggerhead tracks.

| **Environmental Variable** | **Product** | **Spatial Resolution** | **Temporal Resolution** |
| --- | --- | --- | --- |
| Sea-Surface Temperature (SST) | GHRSST/Reynolds OI | 0.25° | 1-day |
| Chlorophyll-a (Chl-a) | SeaWiFS/MODIS | 0.0125° | 8-day |
| Sea-Surface Temperature Root Mean Square (SST RMS) | GHRSST/Reynolds OI | 0.25° | 1-day |
| Magnetic Field Inclination | IGRF -11 | 1.0° | NA |
| Magnetic Field Declination | IGRF -11 | 1.0° | NA |
| Magnetic Field Total Intensity | IGRF -11 | 1.0° | NA |

**STable 2.** Summary of the 65 satellite tracked juvenile loggerhead sea turtles in the North Pacific Ocean that displayed turn around behavior. Turtles were deployed within two regions: the Western North Pacific (Japan) and the Central North Pacific (CNP).

| Track ID | SCL (cm) | Age at Deploy (months) | Captive/Wild Caught | Deploy Date | Deploy  E Lon | Deploy N Lat | Deploy Location | Date Terminated | Distance Traveled (km) | Days Transmitted |
| --- | --- | --- | --- | --- | --- | --- | --- | --- | --- | --- |
| 22208 | 39.7 | 20 | Captive | 24-Apr-2003 | 140.2 | 34.6 | Japan | 24-Sep-2004 | 11,749 | 520 |
| 25359 | 59.4 | 44 | Captive | 24-Apr-2003 | 140.2 | 34.6 | Japan | 12-Jul-2004 | 11,938 | 445 |
| 23045 | 51.4 | 40 | Captive | 28-Nov-2003 | 140.2 | 34.9 | Japan | 21-May-2007 | 22,415 | 1,270 |
| 23486 | 27.4 | 16 | Captive | 28-Nov-2003 | 140.2 | 34.9 | Japan | 22-Jun-2004 | 4,643 | 207 |
| 40649 | 64.8 | 48 | Captive | 23-Apr-2004 | 141.1 | 35.4 | Japan | 31-Dec-2005 | 16,338 | 617 |
| 40651 | 61.9 | 48 | Captive | 23-Apr-2004 | 141.1 | 35.4 | Japan | 4-Jun-2005 | 10,498 | 407 |
| 42718 | 26.6 | 24 | Captive | 23-Apr-2004 | 141.1 | 35.4 | Japan | 8-May-2005 | 11,300 | 380 |
| 50147 | 31.0 | 15 | Captive | 19-Nov-2004 | 140.6 | 34.9 | Japan | 18-Oct-2005 | 9,199 | 334 |
| 50155 | 30.5 | 15 | Captive | 19-Nov-2004 | 140.6 | 34.9 | Japan | 22-Jul-2005 | 8,882 | 245 |
| 24192 | 31.1 | 20 | Captive | 4-May-2005 | 176.6 | 32.7 | Japan | 10-Mar-2007 | 11,637 | 675 |
| 41457 | 35.8 | 19 | Captive | 31-Mar-2009 | 141.4 | 35.4 | Japan | 1-Nov-2009 | 5,256 | 216 |
| 88062 | 37.1 | 19 | Captive | 31-Mar-2009 | 141.4 | 35.4 | Japan | 27-Dec-2009 | 3,965 | 272 |
| 50138 | 36.3 | 20 | Captive | 9-Apr-2010 | 130.5 | 29.7 | Japan | 19-Sep-2011 | 11,536 | 528 |
| 65417 | 37.6 | 20 | Captive | 9-Apr-2010 | 130.5 | 29.7 | Japan | 9-Jun-2011 | 10,799 | 426 |
| 57146 | 32.8 | 20 | Captive | 9-Apr-2010 | 130.5 | 29.7 | Japan | 16-May-2011 | 9,573 | 402 |
| 22278 | 36.0 | 20 | Captive | 9-Apr-2010 | 130.5 | 29.7 | Japan | 17-Jan-2011 | 7,201 | 282 |
| 25360 | 40.7 | 20 | Captive | 9-Apr-2010 | 130.5 | 29.7 | Japan | 15-Jul-2011 | 12,226 | 462 |
| 40719 | 38.1 | 20 | Captive | 9-Apr-2010 | 130.5 | 29.7 | Japan | 23-Feb-2011 | 7,662 | 320 |
| 40725 | 37.3 | 20 | Captive | 9-Apr-2010 | 130.5 | 29.7 | Japan | 27-Feb-2011 | 6,731 | 324 |
| 68336 | 36.9 | 24 | Captive | 12-Jul-2011 | 169.7 | 34.4 | Japan | 26-Apr-2013 | 13,595 | 653 |
| 57150 | 35.1 | 24 | Captive | 12-Jul-2011 | 141.3 | 35.7 | Japan | 14-Oct-2012 | 8,301 | 460 |
| 68335 | 36.2 | 24 | Captive | 12-Jul-2011 | 141.3 | 35.7 | Japan | 17-Oct-2012 | 9,934 | 463 |
| 65421 | 36.5 | 24 | Captive | 12-Jul-2011 | 179.4 | 31.1 | Japan | 20-Apr-2013 | 13,754 | 646 |
| 40471 | 34.7 | 24 | Captive | 12-Jul-2011 | 180.0 | 32.4 | Japan | 4-Feb-2013 | 11,892 | 573 |
| 52697 | 59.4 | 36 | Captive | 12-Jul-2011 | 141.3 | 35.7 | Japan | 27-Sep-2012 | 10,265 | 443 |
| 68330 | 38.6 | 24 | Captive | 12-Jul-2011 | 175.5 | 35.6 | Japan | 17-Mar-2013 | 15,289 | 614 |
| 68331 | 38.2 | 24 | Captive | 12-Jul-2011 | 171.4 | 39.0 | Japan | 18-Feb-2013 | 11,425 | 587 |
| 65433 | 38.9 | 24 | Captive | 12-Jul-2011 | 141.3 | 35.7 | Japan | 2-Aug-2012 | 9,591 | 387 |
| 23002 | 37.4 | 24 | Captive | 12-Jul-2011 | 153.7 | 38.2 | Japan | 23-Nov-2013 | 17,863 | 865 |
| 22535 | 34.9 | 24 | Captive | 12-Jul-2011 | 177.8 | 35.0 | Japan | 28-Feb-2013 | 11,936 | 597 |
| 19595 | 39.1 | 24 | Captive | 12-Jul-2011 | 165.3 | 42.4 | Japan | 14-Sep-2013 | 16,719 | 795 |
| 52693 | 51.8 | 36 | Captive | 12-Jul-2011 | 141.3 | 35.7 | Japan | 28-Jul-2012 | 11,112 | 382 |
| 50146 | 37.9 | 24 | Captive | 12-Jul-2011 | 171.1 | 38.0 | Japan | 15-Jul-2013 | 12,076 | 734 |
| 22270 | 35.4 | 24 | Captive | 15-Jul-2011 | 171.6 | 37.4 | Japan | 2-Aug-2013 | 15,730 | 752 |
| 25359 | 57.5 | NA | Wild | 23-Dec-1998 | 210.0 | 33.6 | CNP | 22-Jul-1999 | 4,426 | 211 |
| 22174 | 51.5 | NA | Wild | 14-Dec-1999 | 209.1 | 32.9 | CNP | 9-Sep-2000 | 5,180 | 271 |
| 22534 | 61.0 | NA | Wild | 19-Aug-2000 | 226.4 | 35.8 | CNP | 1-Feb-2001 | 3,237 | 177 |
| 22329 | 45.5 | NA | Wild | 25-Dec-2002 | 219.1 | 34.7 | CNP | 27-Aug-2003 | 4,464 | 245 |
| 22277 | 43.5 | NA | Wild | 7-Jan-2003 | 216.5 | 32.4 | CNP | 22-Aug-2003 | 3,800 | 226 |
| 19594 | 34.4 | 20 | Captive | 4-May-2005 | 176.6 | 32.7 | CNP | 13-Feb-2008 | 15,759 | 1,014 |
| 50138 | 34.8 | 20 | Captive | 4-May-2005 | 176.6 | 32.7 | CNP | 28-May-2008 | 20,395 | 1,120 |
| 50141 | 37.8 | 20 | Captive | 4-May-2005 | 176.6 | 32.7 | CNP | 19-Oct-2007 | 12,533 | 898 |
| 50136 | 32.7 | 20 | Captive | 4-May-2005 | 176.6 | 32.7 | CNP | 4-Oct-2008 | 18,327 | 1,247 |
| 23177 | 33.4 | 20 | Captive | 4-May-2005 | 176.6 | 32.7 | CNP | 25-May-2008 | 17,224 | 1,117 |
| 50135 | 34.0 | 20 | Captive | 4-May-2005 | 176.6 | 32.7 | CNP | 30-May-2007 | 10,442 | 756 |
| 22534 | 34.2 | 20 | Captive | 4-May-2005 | 176.6 | 32.7 | CNP | 16-Mar-2008 | 18,238 | 1,047 |
| 50133 | 33.1 | 20 | Captive | 4-May-2005 | 176.6 | 32.7 | CNP | 11-Aug-2006 | 8,090 | 463 |
| 23082 | 35.2 | 20 | Captive | 4-May-2005 | 176.6 | 32.7 | CNP | 7-Apr-2008 | 14,447 | 1,069 |
| 57149 | 33.4 | 20 | Captive | 4-May-2005 | 176.6 | 32.7 | CNP | 4-Nov-2006 | 8,563 | 549 |
| 25360 | 35.5 | 20 | Captive | 4-May-2005 | 176.6 | 32.7 | CNP | 29-Nov-2007 | 14,508 | 938 |
| 57148 | 35.8 | 20 | Captive | 4-May-2005 | 176.6 | 32.7 | CNP | 7-Apr-2009 | 25,900 | 1,434 |
| 50142 | 38.4 | 20 | Captive | 4-May-2005 | 176.6 | 32.7 | CNP | 2-Aug-2006 | 7,539 | 455 |
| 25358 | 31.7 | 20 | Captive | 4-May-2005 | 176.6 | 32.7 | CNP | 3-Oct-2007 | 14,752 | 882 |
| 22275 | 36.8 | 20 | Captive | 4-May-2005 | 176.6 | 32.7 | CNP | 10-Jul-2006 | 6,692 | 431 |
| 57142 | 35.9 | 20 | Captive | 4-May-2005 | 176.6 | 32.7 | CNP | 2-Sep-2006 | 6,160 | 486 |
| 57143 | 32.0 | 20 | Captive | 4-May-2005 | 176.6 | 32.7 | CNP | 13-Aug-2006 | 8,364 | 465 |
| 23461 | 35.8 | 20 | Captive | 4-May-2005 | 176.6 | 32.7 | CNP | 29-Jun-2008 | 19,192 | 1,152 |
| 23483 | 31.3 | 20 | Captive | 4-May-2005 | 176.6 | 32.7 | CNP | 22-May-2006 | 6,840 | 383 |
| 23559 | 35.9 | 20 | Captive | 4-May-2005 | 176.6 | 32.7 | CNP | 5-Jan-2008 | 18,641 | 976 |
| 57154 | 35.4 | 20 | Captive | 4-May-2005 | 176.6 | 32.7 | CNP | 15-Oct-2006 | 9,410 | 529 |
| 52695 | 34.0 | 20 | Captive | 4-May-2005 | 176.6 | 32.7 | CNP | 23-Apr-2006 | 5,171 | 354 |
| 57145 | 36.1 | 20 | Captive | 4-May-2005 | 176.6 | 32.7 | CNP | 20-Nov-2006 | 12,170 | 565 |
| 57152 | 36.6 | 20 | Captive | 4-May-2005 | 176.6 | 32.7 | CNP | 27-Jun-2006 | 7,323 | 419 |
| 57144 | 34.9 | 20 | Captive | 4-May-2005 | 176.6 | 32.7 | CNP | 31-Jul-2006 | 8,376 | 452 |
| 65429 | 28.0 | NA | Captive | 27-Oct-2006 | 176.8 | 32.9 | CNP | 24-May-2007 | 4,118 | 208 |

**STable 3.** Model selection results from generalized additive modeling of the environmental conditions at the location of reversal in migration (‘turnaround’) versus the conditions experienced by a turtle as it continued to move eastward across the North Pacific Ocean. Presented are the p-values, r-squared, estimated degrees of freedom, and AIC for each of the eleven environmental parameters for (a) Japan deployed turtles and (b) turtles deployed within the Central North Pacific.

a)

| **Deploy Region:** | **Japan** |  |  |  |  |
| --- | --- | --- | --- | --- | --- |
| **Explanatory Variable** | **R-squared** | **AIC** | **Dev Exp** | **Estimated DF** | **P-value** |
| SST | 0.05 | 94.72 | 6.2 | 2.15 | 0.23 |
| Chl | 0.11 | 82.04 | 12.58 | 2.88 | 0.1 |
| SST RMS | 0.08 | 92.12 | 8.94 | 2.3 | 0.15 |
| Inclination | 0.02 | 96.37 | 3.08 | 1.5 | 0.49 |
| Declination | 0.17 | 87.13 | 16.48 | 3.2 | < 0.01** |
| Month | 0.03 | 95.17 | 3.8 | 1.24 | 0.18 |
| nDays | 0.13 | 88.9 | 10.74 | 1.38 | < 0.01** |
| SCL | 0 | 96.27 | 0 | 0 | 1 |

b)

| **Deploy Region:** | **Central North Pacific** | |  |  |  |
| --- | --- | --- | --- | --- | --- |
| **Explanatory Variable** | **R-squared** | **AIC** | **Dev Exp** | **Estimated DF** | **P-value** |
| SST | 0.02 | 90.87 | 2.87 | 1.35 | 0.54 |
| Chl | 0.02 | 78.74 | 4.02 | 1.63 | 0.56 |
| SST RMS | 0.04 | 87.89 | 5.22 | 1.89 | 0.24 |
| Inclination | 0.17 | 80.7 | 14 | 1.2 | < 0.01** |
| Declination | 0.04 | 89.38 | 5.24 | 1.65 | 0.13 |
| Month | 0.42 | 65.76 | 40.1 | 5.31 | < 0.05* |
| nDays | 0.03 | 89.42 | 2.96 | 0.66 | 0.11 |
| SCL | 0 | 90.72 | 0 | 0 | 1 |

**Supplemental Figures**

**
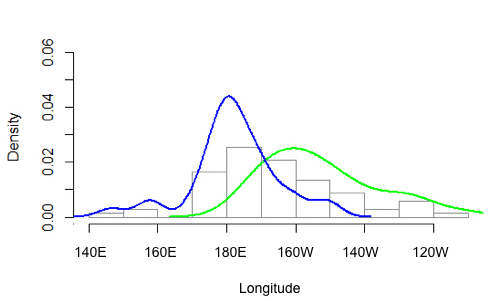
SFigure 1.** Density histogram of maximum eastward longitude for (a) all turtles, n=231 (gray), long-term tracks deployed off Japan, n= 34 (blue line), and long-term deployments in the Central North Pacific, n=31 (green line).

**SFigure 2.** Examples of east-west movement in individual long-term tracks. Example of 2 turtles deployed in the western (a) and (b) central North Pacific that reached a maximum eastward trajectory and changed dominant direction. Panels c and d show each track moving in an east-west-east direction, with the start location designated by a blue triangle and final location designated with a red square. Both tracks initially moved eastward (gray line) and then reversed direction, moving westward (blue segment of track). Both changed dominant direction for second time, once again moving eastward (green segment of track). Track ‘68330’ transmitted for 614 days (15,289 km). Track ‘22534’ transmitted for 1,047 days (18,238 km).

c)
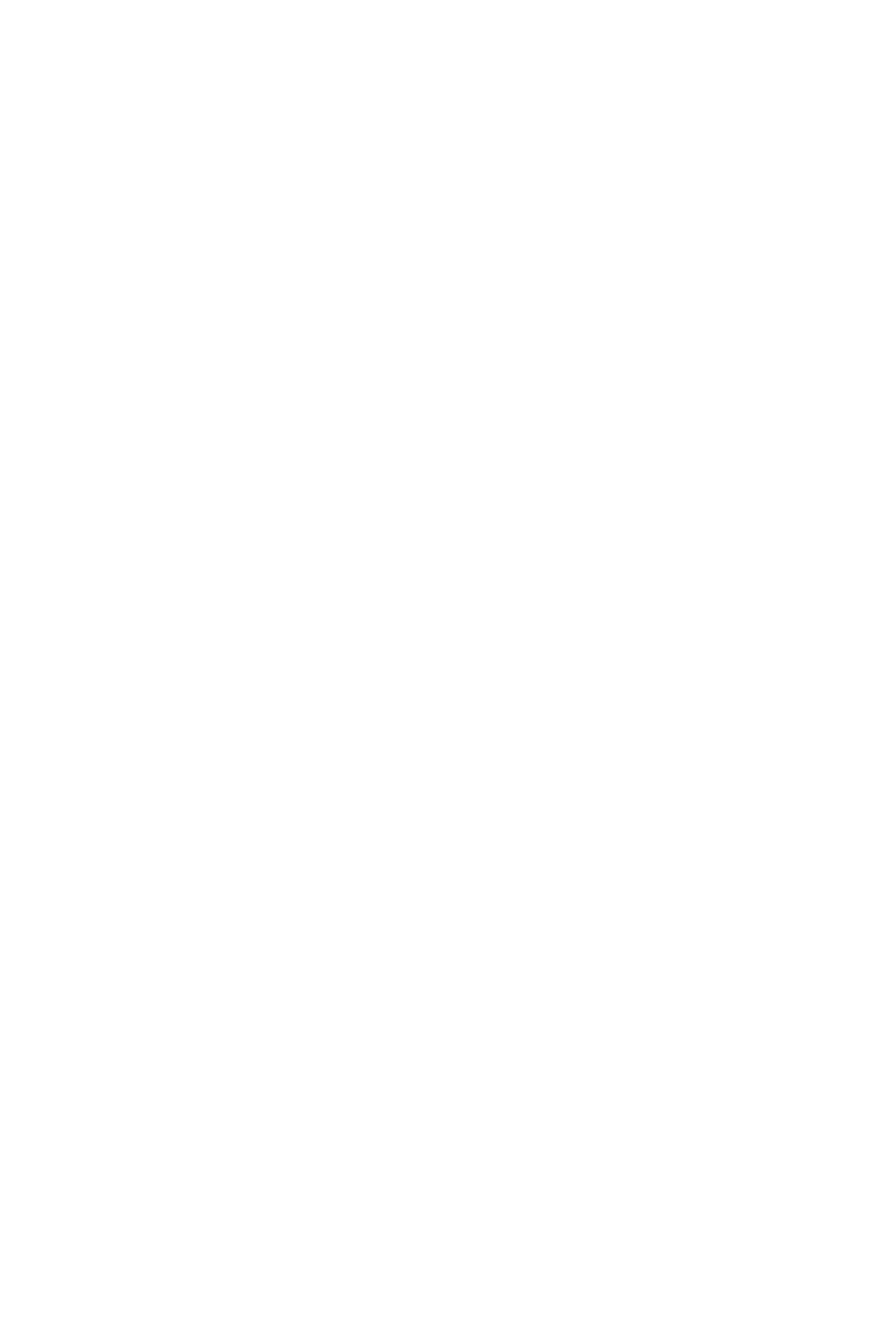
)

a)

b)

d)
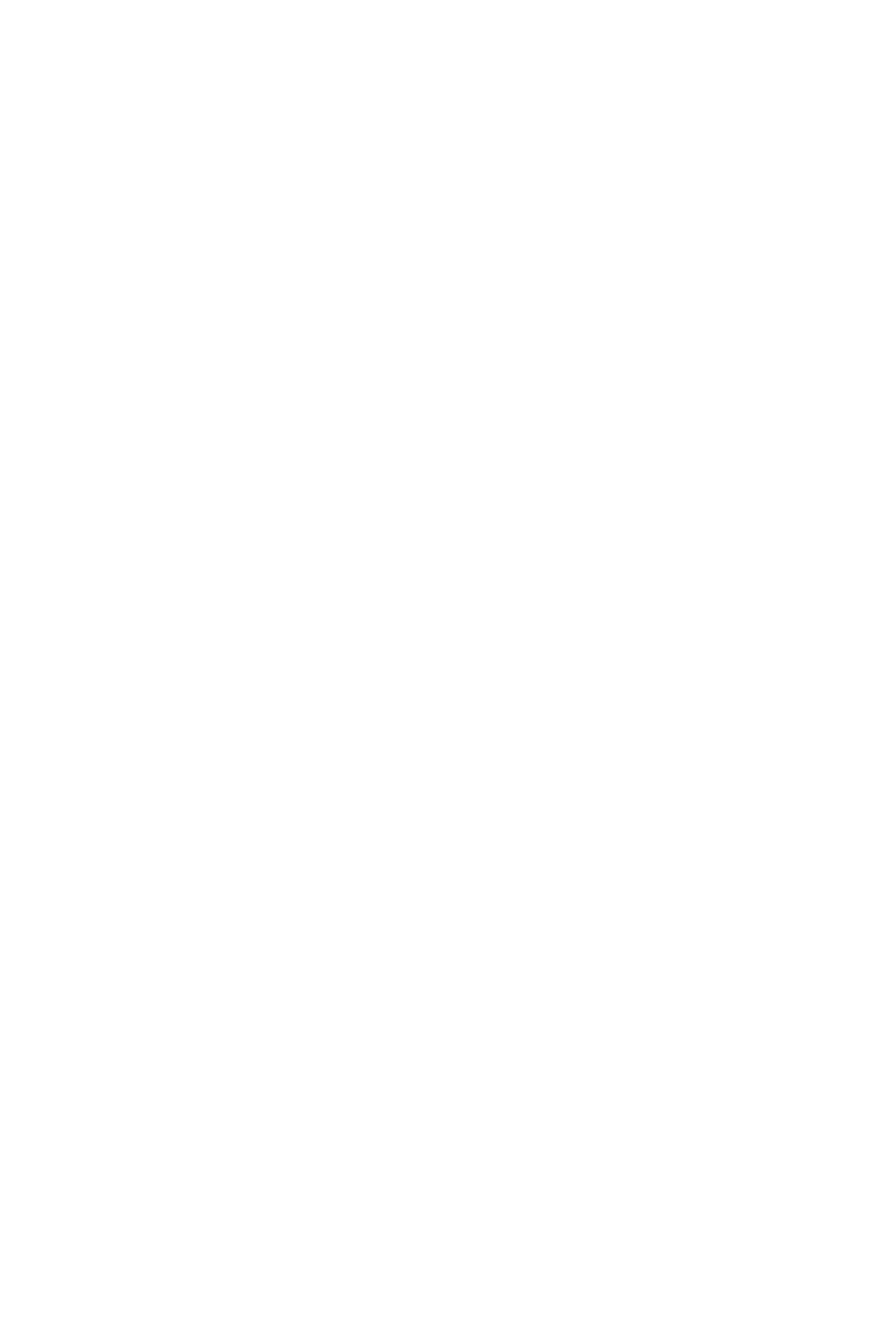
)

**
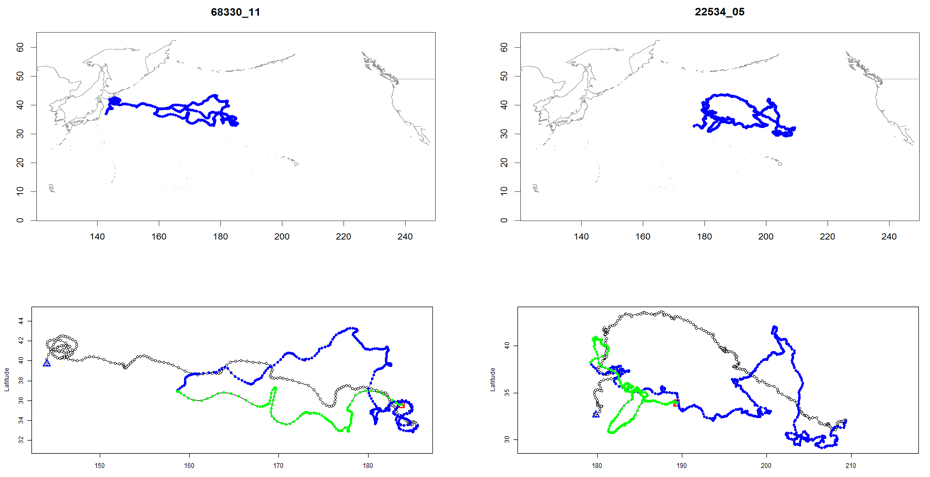
**

**SFigure 3.** (a) Average sea-surface temperature (SST °C) and (b) Chlorophyll-a concentrations (mg m^-3^) for the North Pacific Ocean basin, from 1997 - 2014)


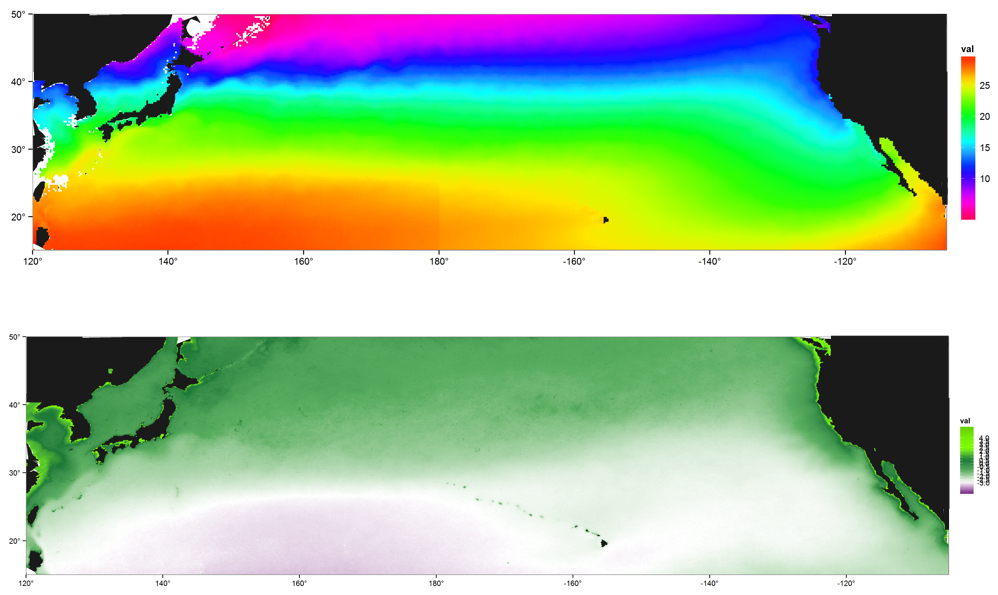


a)

b)

**SFigure 4.** GAM response curves of juvenile loggerhead east-west movements. Positive values show the likelihood of an animal reversing direction under a range of environmental values. Results indicate that turtles deployed off Japan are more likely to reverse direction with an increase in declination (a) and the longer it travels since deployment (b). For the turtles deployed in the Central North Pacific, turtles are more likely to reverse direction under lower values of magnetic field inclination (c) and during the first half of the year (d). Gray shading represents the 95% confidence intervals for the fitted relationships.


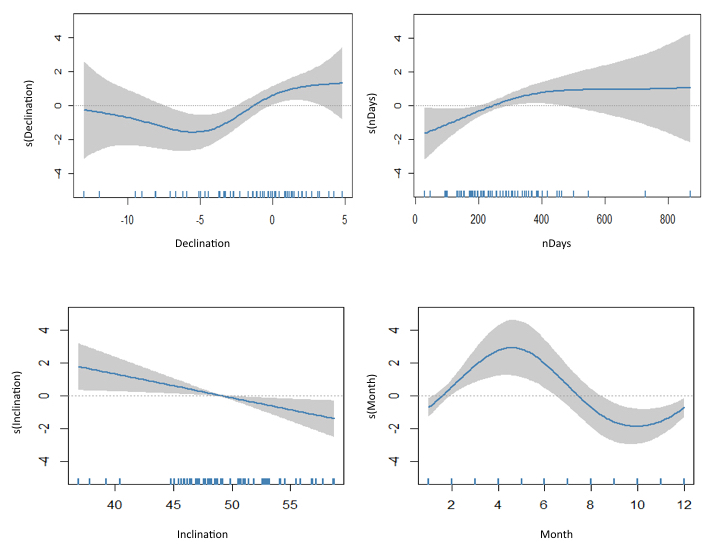


Likelihood of continuing to move eastward

Likelihood of reversing direction (east 🡪 west)

d)

c)

b)

a)
